# The effects of genetic variation and environmental factors on rhynchophylline and isorhynchophylline in *Uncaria macrophylla* Wall. from different populations in China

**DOI:** 10.1371/journal.pone.0199259

**Published:** 2018-06-28

**Authors:** Mei-Fang Song, Yan-Hong Guan, Hai-Tao Li, Shu-Gen Wei, Li-Xia Zhang, Zhong-Lian Zhang, Xiao-Jun Ma

**Affiliations:** 1 The Key Laboratory of Dai and Southern Medicine of Xishuangbanna Dai Autonomous prefecture, Yunnan Branch of Institute of Medicinal Plant Development, Chinese Academy of Medical Sciences, Peking Union Medical College, Jinghong, China; 2 Guangxi Botanical Garden of Medicinal Plants, Nanning, China; 3 Institute of Medicinal Plant Development, Chinese Academy of Medical Sciences, Peking Union Medical College, Beijing, China; Sudbury Regional Hospital, CANADA

## Abstract

*Uncaria macrophylla* Wall. is an important Chinese medicinal herb. Rhynchophylline (RIN) and isorhynchophylline (IRN) are its major active compounds. We investigated the influence of genetic differentiation and environmental factors on the RIN and IRN to find the main influencing factors of their contents and lay the foundation for the following cultivation and breeding. We used inter-simple sequence repeat (ISSR) markers to investigate the genetic diversity, and high-performance liquid chromatography (HPLC) to measure the contents of RIN and IRN in 200 samples of *U*. *macrophylla* obtained from nine natural populations, and then to analyze the correlation between genetic differentiation, environmental factors of sampling sites and the contents of RIN and IRN. We found that High intra-population (80.05%) and low inter-population (19.95%) genetic diversity existed in the samples of *U*. *macrophylla*. To some extent, genetic differentiation and the contents of RIN and IRN had correlation in individual populations (such as JH, MH, XM, and ML). The RIN and IRN contents were significant negatively correlated with the precipitation in May (R_IRN_ = -0.771, *p* = 0.015) and June (R_RIN_ = -0.814, *p* = 0.008; R_IRN_ = -0.921, *p* = 0.000), indicating that precipitation was the main affecting factor of their contents. Interestingly, the analysis results showed that the RIN content had a significant positive correlation (r = 0.585, *p* = 0.000) with the IRN content (they are isomers); the proportion of RIN had a significant negative correlation with the sum of the two (r = –0.390, *p*<0.0001), while the proportion of IRN had a significant positive correlation (r = 0.390, *p*<0.0001). It meant that, with the total quantity of the two compounds increased, the proportion of RIN decreased and the proportion of IRN increased, illustrating that their conversion exist some regularity. Moreover, the content ratio of RIN and IRN was significant positively correlated with the January precipitation (r = 0.716, *p* = 0.030), implying that January may be the key period for the mutual transformation of RIN and IRN.

## Introduction

*Uncaria macrophylla* Wall., a medicinal plant belonging to the family Rubiaceae, is one of the main original plant of Uncariae Ramulus cum Uncis (known as “Gou-teng” in China), as recorded in the Pharmacopoeia of the People’s Republic of China [1]. In China, *U*. *macrophylla* grows mainly in Yunnan and Guangxi Provinces [2]. Gou-teng is a traditional Chinese medicine with RIN and IRN as its main active compounds [3, 4]. It is used to treat hypertension, cardiac arrhythmias, depression, and Alzheimer’s disease and so on [5–9]. However, our previous study showed that there was a great divergence in the contents of RIN and IRN in *U*. *macrophylla* from various regions and the difference between the highest and the lowest was nearly 10 times. Huang et.al also got the similar results [10]. The above phenomenon indicated that the quality uniformity of *U*. *macrophylla* is poor, which was bound to seriously affect its clinical efficacy. Therefore, seeking the main factors affecting the contents of RIN and IRN, and then purposefully improving their content and uniformity is necessary to solve the above problems.

It was known that the content of active ingredients was influenced by both genetic factors and ecological environment. In this paper, the genetic diversity of *U*. *macrophylla* was analyzed by ISSR and the contents of RIN and IRN by HPLC in order to analyze the effect of genetic factors on the content of active components; Furthermore, the ecological factors (including precipitation, temperature and soil factors) of sampling point were collected, and the correlations between the content of main active components (RIN and IRN) and various ecological factors were analyzed in order to screen key ecological factors affecting the contents of RIN and IRN.

By analyzing the relationships between population genetic diversit, environmental factors and the contents of RIN and IRN, we can confirm the influence of biotic and abiotic factors on the contents of RIN and IRN and propose appropriate methods for cultivated location selection and germplasm screening to improve the content of active ingredients and ensure the quality of“Gou Teng” medicinal materials.

## Materials and methods

### Ethics statement

All specimens were collected at locations for which specific permission for entry was not required. None of these locations were protected areas or private land. *Uncaria macrophylla* Wall. is not currently a protected species in China; therefore, no specific permission was required to conduct the fieldwork.

### Plant materials and environmental data sampling

**Fig 1:**
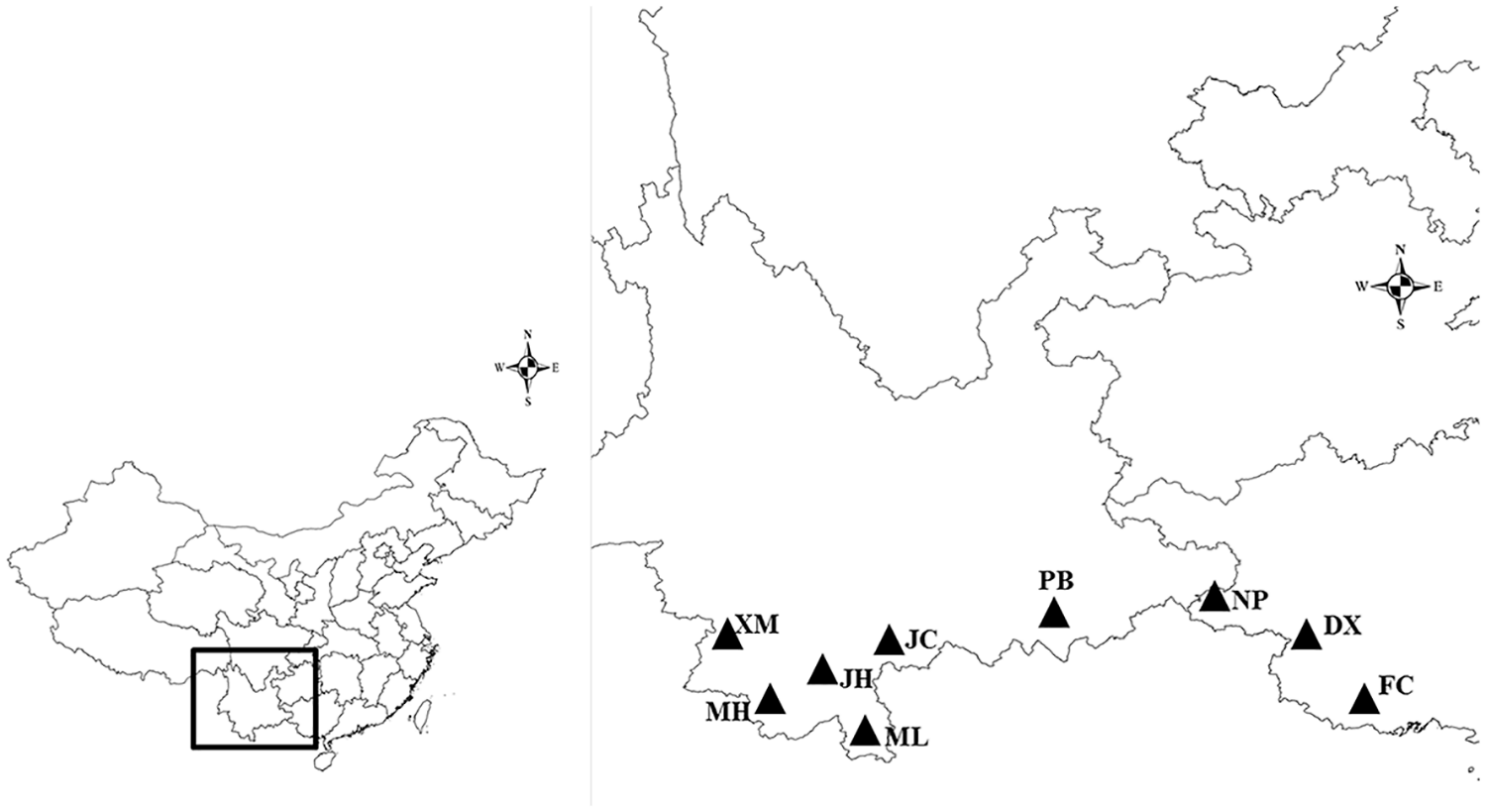
Distribution of the sampling location.

According to the Pharmacopoeia of the People’s Republic of China, Gou-teng medical material is harvested from September to October. Therefore, we sampled 200 individuals from nine populations across the main range of *U*. *macrophylla* from early September to early October, 2016 for chemical examination and genetic analyses (Fig 1). A random sample of 15–30 individuals was obtained from each population. The meteorological data for the nine populations, including monthly precipitation, monthly temperature, and seasonal precipitation, were obtained from local meteorological stations (Table 1 and S1 Table) and the distribution area information (including latitude, longitude, altitude and soil texture) were gathered during the sampling (Table 1 and S2 Table).

**Table 1:** The sampling information for 9 populations.

population	Province	county	Sample size	latitude(°)	longitude(°)	Altitude(m)	Variable coefficient of seasonal precipitation	Variation range of mean annual temperature(°C)	Mean annual precipitation(mm)	Mean annual temperature(°C)
NP	Guangxi	Napo	20	22.95~23.13	105.67~106.00	320~865	84.80	21.63	1459.00	20.66
DX	Guangxi	Daxin	15	22.84~22.89	106.74~106.78	334~512	79.27	22.67	1430.87	21.42
FC	Guangxi	Fangcheng	20	22.15~22.70	103.76~106.75	140~490	72.32	22.13	1260.55	22.67
PB	Yunnan	Pingbian	15	22.98~23.01	103.74~103.76	474~1135	80.40	19.49	1310.67	20.14
JC	Yunnan	Jiangcheng	30	22.41~22.78	101.56~102.07	895~1303	82.73	21.22	1827.33	19.87
ML	Yunnan	Mengla	30	21.32~22.31	101.48~101.62	665~1221	78.17	21.50	1616.87	20.93
XM	Yunnan	Ximeng	20	22.60~22.70	99.56~99.66	739~1440	83.95	24.48	1438.15	21.01
MH	Yunnan	Menghai	17	21.56~22.33	100.02~101.00	1037~1374	84.11	23.59	1499.44	19.74
JH	Yunnan	Jinghong	30	21.57~22.38	100.35~101.01	1002~1367	81.43	22.90	1509.33	19.45

### High-performance liquid chromatography

Individual specimens were dried in the shade at room temperature. Each dried sample was pulverized, and about 3 g power was weighed accurately and then extracted with 60 ml 100% methanol for 60 min in an ultrasonic bath, during which ice was added several times to keep the water temperature about 4 °C. After adding the loss weight, the extracts were filtered through a 0.45-μm membrane prior to HPLC analysis and determined within 24h. The chromatographic separation was performed on a Waters 600 HPLC system (Waters, USA), equipped with a Waters 2998 photodiode array detector, Waters 600 pump, Waters 2707 autosampler, and Waters in-line degasser AF. A Waters XTerra RP-18 column (4.6 × 250 mm, 5 μm) was also used. The column was kept at 35°C and the flow rate was 1 mL/min. The photodiode array detector was set at 245 nm. Methanol and H_2_O (0.01 M triethylamine, pH 7.5) were used in the mobile phase according to a 55%:45% isocratic elution program.

### Chemical data analysis

Principal component analysis (PCA) was used to examine the relative distributions of different populations of *U*. *macrophylla* according to their chemical concentration data (mean values of individuals from each population) (Table 2). Agglomerative hierarchical clustering (AHC) analysis was used to construct a dendrogram based on the distances among the population chemical content data (mean values). The RIN and IRN contents, in addition to their ratio, sum, and respective proportions, were analyzed using the correlation test in XLSTAT 2015 software (Addinsoft, France) according to the chemical content data obtained from 200 individual samples (S3 Table).

**Table 2:** The contents of chemical compounds in 9 populations.

population	RIN%			IRN%		
	mean	maximum	Minimum	mean	maximum	Minimum
NP	0.1697±0.0423	0.2386	0.0961	0.0589±0.0317	0.1558	0.0170
DX	0.1966±0.0355	0.2634	0.1402	0.0557±0.0234	0.1054	0.0157
FC	0.2239±0.0507	0.3245	0.1333	0.0631±0.0185	0.1034	0.0349
PB	0.2210±0.0507	0.3178	0.1519	0.0664±0.0301	0.1246	0.0293
JC	0.1806±0.0374	0.2616	0.0940	0.0496±0.0200	0.0948	0.0187
ML	0.2069±0.0493	0.3458	0.1483	0.0657±0.0360	0.2130	0.0218
XM	0.1906±0.0398	0.2750	0.1401	0.0600±0.0207	0.0977	0.0297
MH	0.2039±0.0385	0.2705	0.1505	0.0626±0.0255	0.1294	0.0289
JH	0.2120±0.0431	0.3241	0.1365	0.0643±0.0223	0.1217	0.0235

### Isolation of DNA and ISSR analysis

Total genomic DNA was isolated from silica gel-dried leaves following the manufacturer’s instructions for the Plant Genomic DNA Kit (DP305 Tiangen Biotech, China). The quality and quantity of the isolated genomic DNA was assessed by electrophoresis on 1.0% agarose gels, staining with ethidiumbromide and comparing with a set of known DNA concentration standards, and UV spectroscopy using a NanoDrop Spectrophotometer.

Sixty ISSR primers were tested (published by Columbia University), from which only the ten primers that produced relatively more repeatable and distinct fragments were selected. ISSR amplification was performed in a 20 μl volume containing 10–30 ng of genomic DNA template, 1×PCR buffer with 1.5 mM MgCl2, 0.2 mM of each dNTP, 0.5 μM of each primer (synthesized by Life Technologies, China), and 1.5 U Taq DNA Polymerase (DR001; Takara, China). The amplifications were carried out for an initial 4 min at 94°C, followed by 30 cycles of 45 s at 94°C, 45 s at the respective annealing temperature (55–58°C), 2 min at 72°C, and a final 10-min extension at 72°C. The amplification products were electrophoresed in 2% agarose gels and stained with ethidium bromide. The gels were visualized and photographed using the UVItec system (Alpha Innotech, USA). To confirm the reproducibility of the banding patterns, the PCR experiments were repeated twice.

### Genetic data analysis

Data were recorded in terms of the presence (1) or absence (0) of a band. Only distinct, well-separated bands were included in the analysis. To assess genetic diversity, Nei’s gene diversity index (H), the Shannon index (I), percentage of polymorphic loci (PPL), population diversity (HS), total gene diversity (HT) within populations, and inter-population differentiation (GST) were calculated using the POPGENE 1.32 program [11,12]. Genetic similarities according to Jaccard’s coefficient were calculated using the SIM-QUAL function in the numerical taxonomy multivariate analysis system NTSYSpc (ver. 2.10) [13] and dendrograms were constructed with the SAHN clustering program using the unweighted pair group method with arithmetic means (UPGMA).

### Combined data analysis

To eliminate the effects of different scales of measurement, the data were standardized using SPSS software (ver. 16.0; SPSS Inc, USA). The contents of the two active components measured by HPLC, and the environmental data gathered from the meteorological stations and field work, were combined and analyzed using correlation tests in XLSTAT 2015 to detect the key factors affecting the contents of the active compounds. The genetic UPGMA tree constructed based on genetic distances among different populations, and the chemical dendrogram constructed according to the distance among the population chemical contents, were compared to determine the correlation between genetic and chemical differentiation.

## Results

### Chemical components and correlation analysis

**Fig 2:**
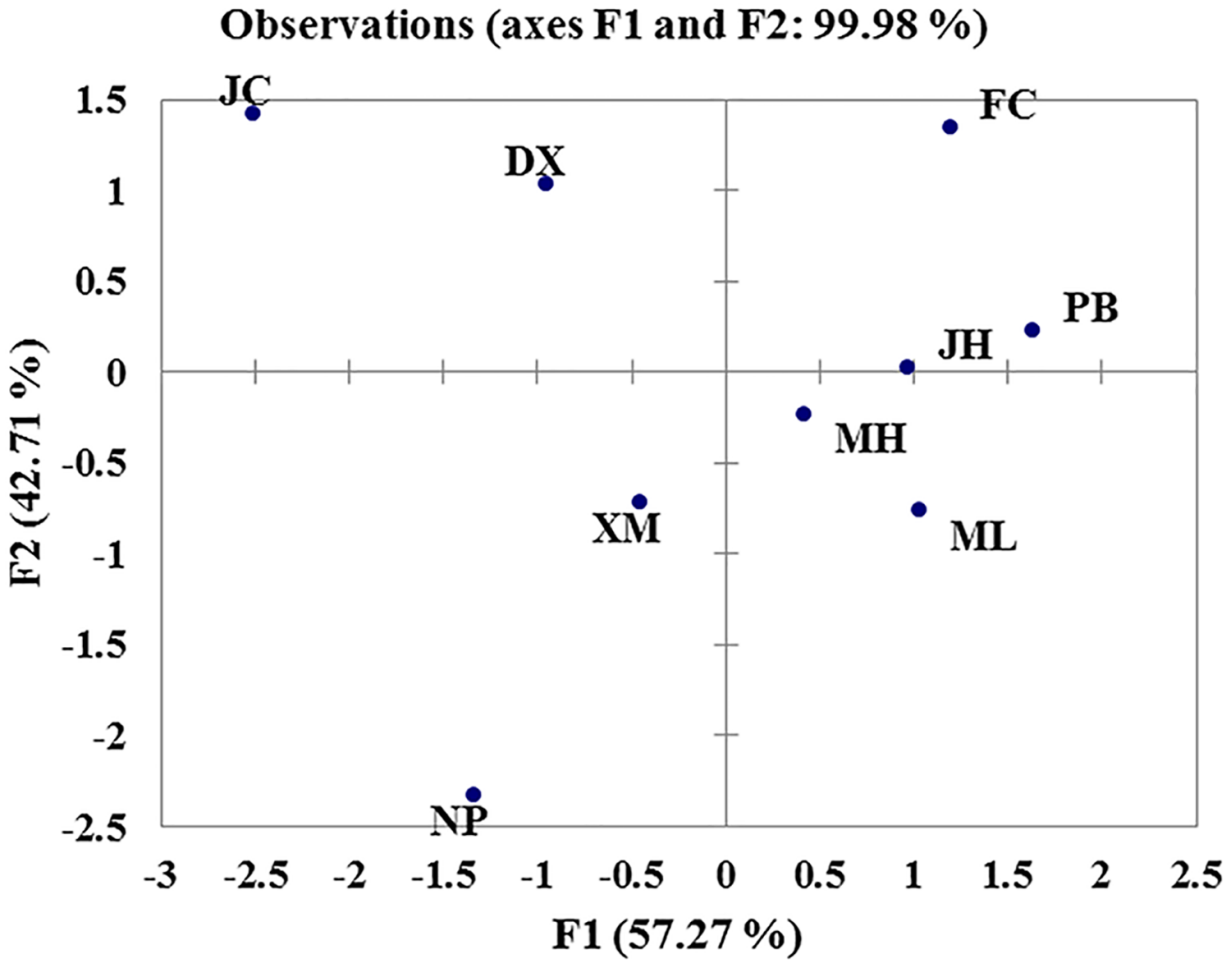
The PCA distribution of different populations.

**Fig 3:**
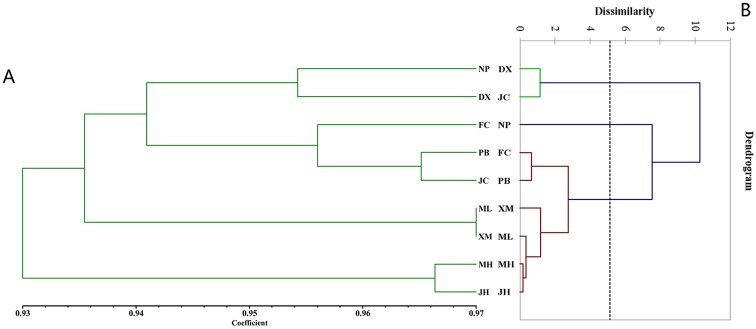
Comparison between genetic dendrogram (A) and chemical dendrogram (B).

Among the 200 individuals, the RIN content varied from 0.9mg/g to 3.5mg/g and the IRN content from 0.2mg/g to 2.1mg/g (S3 Table). Based on the comparisons of the maximum, minimum, and mean values of the RIN and IRN contents in *U*. *macrophylla* of the nine populations, we discovered that this broad range was mainly within rather than among the populations (Table 2). The PCA distribution plot of the populations based on the chemical characteristics showed that the population JH, MH, ML, XM, and PB clustered together, while the population JC, DX, FC and NP were more scattered (Fig 2). The AHC dendrogram based on chemical components contents divided the nine populations into three clades: Clade 1 (population JH, MH, ML, XM, PB, and FC), Clade II (population DX and JC), and Clade III (population NP) (Fig 3B). It can be seen that the clustering results obtained with the above two methods were essentially consistent. To probe biogeographic connections, we compared the clustering results with the sampling map (Fig 1). Interestingly, the chemical cluster of the population JH, MH, ML, XM, and PB obviously matched their geographic distribution, indicating that the content of active compounds in *U*. *macrophylla* from these populations has a significant association with geographic location.

To assess the transformation between RIN and IRN, we analyzed the correlations among the RIN and IRN contents, their sum, and the respective proportion. The results indicated that RIN content had a significant positive correlation with IRN content (r = 0.585, *p* = 0.000); the proportion of RIN had a significant negative correlation with the sum of the two compounds (r = –0.390, *p*<0.0001), while the proportion of IRN had a significant positive correlation (r = 0.390, *p*<0.0001) (Table 3).

**Table 3:** Results of correlation test based on chemical components content in 200 individuals.

Variables	RIN%	IRN%	Sum(RIN+IRN)	RIN/Sum%	IRN/Sum%
RIN%	**1**	**0.000**	**0.000**	0.246	0.246
IRN%	**0.585**	**1**	**<0.0001**	**<0.0001**	**<0.0001**
Sum(RIN+IRN)	**0.945**	**0.818**	**1**	**<0.0001**	**<0.0001**
RIN/Sum%	-0.082	**-0.823**	**-0.390**	**1**	**<0.0001**
IRN/Sum%	0.082	**0.823**	**0.390**	**-1.000**	**1**

Values in bold are different from 0 with a significance level, alpha = 0.05.

Correlation matrix (pearson) is listed below the diagonal, while the *p*-values are listed above the diagonal.

### Genetic data analysis

The identified 10 ISSR primers screened from the original 60 primers were used in the genetic diversity analysis. The number of bands amplified with each primer ranged from 16 to 26 (average of 20). The survey of 200 individuals from the nine *U*. *macrophylla* populations produced a total of 201 amplified bands, of which 195 were polymorphic (97.01%) (S4 Table). The highest genetic diversity was observed in population JC (H = 0.2031 ± 0.1799, I = 0.3167 ± 0.2529) and ML (H = 0.2106 ± 0.1812, I = 0.3266 ± 0.2545), and their proportion of polymorphisms was all 75.12%. By contrast, the lowest genetic diversity was observed in population DX (H = 0.1752 ± 0.1827, I = 0.2723 ± 0.2611) and the proportion of polymorphisms was 62.19% (S5 Table). The total Nei’s genetic diversity of all populations (HT) was 0.2417 ± 0.0261; while the Nei’s genetic diversity within populations (HS) was 0.1935 ± 0.0173. In addition, the Nei’s gene differentiation coefficient (GST) was 0.1995. The high value of NM (2.0058) indicated that gene flow among populations of *U*. *macrophylla* was quite frequent, which meant that the majority of the genetic variation occurred within populations, while the genetic differentiation among populations was not significant.

To represent the genetic relationship among populations, an UPGMA dendrogram was constructed based on their genetic distances (Fig 3A). The dendrogram showed that the genetic diversity of populations followed their regional distribution, i.e., the populations that clustered together (e.g., JH and MH; XM and ML; JC and PB) were also close together geographically.

### The relationship between genetic and chemical variation

To some extent, a few populations (such as JH, MH, XM and ML) had correlation between genetic differentiation and the contents of RIN and IRN, while the most populations had no correspondence (Fig 3). In other words, the RIN and IRN contents in Gou-teng medicinal material obtained from these populations may be more affected by genetic differentiation than other populations.

### The relationship between environmental factors and chemical differentiation

The correlation tests indicated that the content of RIN had significant negative correlation with Variable coefficient of seasonal precipitation (r = -0.693, *p* = 0.038) (S6 Table), indicating the content of this compound was sensitive to the varied range of precipitation. Further analysis indicated that the contents of RIN and IRN had significant negative correlations with the precipitation in May (R_IRN_ = -0.771, *p* = 0.015) and June (R_RIN_ = -0.814, *p* = 0.008; R_IRN_ = -0.921, *p* = 0.000), while the content ratio of these two compounds was significant positively correlated with the January precipitation (r = 0.716, *p* = 0.030) (Table 4). The RIN and IRN contents had no relationship with any other environmental factor (S7 and S8 Tables).

**Table 4:** The correlation between chemical compounds and monthly precipitation in 9 populations.

Variables	January	February	March	April	May	June	July	August	September	October	November	December
RIN%	0.128 (0.743)	0.109 (0.780)	0.110 (0.777)	0.084 (0.831)	-0.657 (0.054)	**-0.814** **(0.008)**	-0.481 (0.190)	-0.562 (0.115)	-0.183 (0.637)	-0.149 (0.703)	-0.158 (0.684)	0.013 (0.974)
IRN%	-0.404 (0.281)	-0.170 (0.662)	-0.262 (0.497)	-0.218 (0.572)	**-0.771** **(0.015)**	**-0.921** **(0.000)**	-0.497 (0.173)	-0.398 (0.289)	-0.133 (0.732)	-0.042 (0.915)	-0.065 (0.869)	-0.097 (0.804)
RIN/IRN%	**0.716** **(0.030)**	0.352 (0.353)	0.494 (0.177)	0.399 (0.288)	0.182 (0.640)	0.181 (0.642)	0.083 (0.831)	-0.161 (0.679)	-0.018 (0.964)	-0.102 (0.794)	-0.073 (0.852)	0.191 (0.623)

Values in bold are different from 0 with a significant level, alpha = 0.05 and *p*-values are listed in parentheses.

## Discussion

Genetic diversity studies of *U*. *macrophylla* could reveal the genetic variation within and among populations, and illuminate the genetic structure of *U*. *macrophylla*. The value of Nei’s GST was 0.1995, indicating that 19.95% of the genetic variation was among populations and 80.05% was within populations, which meant that genetic differentiation mainly occurred within, and rarely among, the populations. *U*. *macrophylla* is a perennial woody liana with a wide distribution in the wild. Its seeds are very small and scatter easily and randomly after falling, which leads to frequent gene flow among populations. Therefore, genetic differentiation has rarely occurred among the populations. Furthermore, the high genetic variation within the populations indicated that *U*. *macrophylla* has strong adaptability to environmental changes and strong evolutionary potential, in turn indicating that many individual plants within each population should be collected as a germplasm resource for *U*. *macrophylla*.

The cluster graphs of genetic differentiation and chemical composition content showed that individual populations could be clustered into a class both in the two cluster graphs, while the clustering positions of the most populations in the two cluster graphs were not corresponding to each other, indicating that the contents of RIN and IRN in *U*. *macrophylla* was influenced by both genetic factors and environmental factors. The results suggest that we should not only consider the influence of germplasm resources on the quality of medicinal materials, but also the influence of environmental factors present at cultivation locations to obtain medicinal materials containing high levels of active compounds.

The RIN and IRN contents of the samples in this study were negatively correlated with the precipitation in May and June, i.e., the higher the rainfall, the lower the contents of the two compounds. It is well known that alkaloids are secondary metabolites produced by plants to adapt to the adverse environment. The above results may be attribute to that May and June are the critical period for vegetative growth of *U*. *macrophylla*, in which the greater the precipitation, the more conducive to its growth, making it in a non-adversity state, and further retarding the biosynthesis of RIN and IRN. This suggests that areas with low rainfall could harvest the high quality *U*. *macrophylla* materials. Besides, RIN and IRN is a pair of diastereoisomers that can transform mutually [14, 15]. The content ratio of RIN and IRN was positively correlated with the January precipitation, implying that January may be the key period for conversion of the two compounds.

Previous studies had shown that the targets and strength of pharmacological effect differ between RIN and IRN in Gou-teng: IRN has a relatively strong antihypertensive effect, while RIN has relatively strong effects on the nervous system [16–18]. The two isomers can transform into each other under certain conditions [14, 15]. Our determination of the compounds contents in 200 individuals indicated that RIN content had a significant positive correlation with that of IRN; moreover the proportion of RIN had a significant negative correlation with the summed amount of the two alkaloids, while the proportion of IRN had a significant positive correlation with the sum. These results indicated that, with the amount of RIN increased, that of IRN increased simultaneously, such that the overall amount of the two alkaloids also increased; however, the proportion of RIN decreased relative to that of IRN. The status illustrated that some of RIN had turned into IRN. The results showed that the content ratio of RIN and IRN was positively correlated with the January precipitation, that is, January precipitation may be a key factor affecting the conversion rate between RIN and IRN. The proportions of RIN and IRN in Gou-teng medicinal materials directly affect their clinical application. Therefore, it is important to produce Gou-teng medicinal materials that are appropriate for treating different illnesses according to the transform rule.

## Supporting information

S1 Table Monthly precipitation (temperature) information for 9 populations unit: mm(°C). (DOCX)

S2 Table The soil texture information for 9 populations. (DOCX)

S3 Table The information of chemical compounds from 200 individuals. (DOCX)

S4 Table ISSR primer screening. (DOCX)

S5 Table Genetic diversity index of the 9 populations. (DOCX)

S6 Table The correlation between chemical compounds and other environment indexes in 9 populations. (DOCX)

S7 Table The correlation between chemical compounds and monthly temperature in 9 populations. (DOCX)

S8 Table The correlation between chemical compounds and soil texture in 9 populations. (DOCX)

## Abbreviations

AHC: Agglomerative hierarchical clustering
HPLC: high-performance liquid chromatography
IRN: isorhynchophylline
ISSR: inter-simple sequence repeat
PCA: Principal component analysis
RIN: rhynchophylline
UPGMA: unweighted pair group method with arithmetic means
