# Supplementary material for: The effects of genetic variation and environmental factors on rhynchophylline and isorhynchophylline in Uncaria macrophylla Wall. from different populations in China
Source: PLoS One. 2018 Jun 28;13(6):e0199259. doi: 10.1371/journal.pone.0199259 (PMC6023176; doi:10.1371/journal.pone.0199259)
Supplement: S1 Table — (DOCX) [file pone.0199259.s001.docx]

**S1 Table****. Monthly precipitation（temperature）information for 9 populations unit: mm(℃)**

| population | January | February | March | April | May | June | July | August | September | October | November | December |
| --- | --- | --- | --- | --- | --- | --- | --- | --- | --- | --- | --- | --- |
| NP | 12.15  （12.49） | 30.05  （14.34） | 29.65  （17.94） | 72.45  （21.88） | 183.05  （24.79） | 260.60  （25.95） | 281.80  （26.47） | 294.05  （26.08） | 147.50  （24.56） | 87.75  （21.40） | 41.85  （17.63） | 18.10  （14.27） |
| DX | 23.13  （12.99） | 40.60  （14.45） | 32.93  （18.23） | 76.20  （22.37） | 195.00  （25.85） | 267.87  （27.04） | 257.20  （27.53） | 257.07  （27.17） | 136.00  （25.75） | 86.80  （22.36） | 37.67  （18.49） | 20.40  （14.85） |
| FC | 19.55  （14.56） | 35.68  （15.88） | 38.91  （19.25） | 85.41  （23.29） | 157.09  （27.08） | 196.18  （28.23） | 208.09  （28.43） | 232.27  （28.11） | 152.73  （26.98） | 80.32  （23.76） | 33.18  （19.99） | 21.14  （16.43） |
| PB | 11.80  （13.31） | 30.80  （14.84） | 33.87  （18.41） | 74.20  （21.72） | 157.13  （23.90） | 214.00  （24.62） | 274.00  （24.79） | 239.73  （24.56） | 128.00  （23.33） | 80.80  （20.51） | 49.53  （17.30） | 16.80  （14.37） |
| JC | 21.00  （14.04） | 26.40  （15.47） | 38.03  （18.37） | 80.07  （21.25） | 191.00  （23.07） | 295.47  （23.58） | 400.27  （23.48） | 337.43  （23.51） | 201.70  （22.73） | 127.87  （20.74） | 76.40  （17.61） | 31.70  （14.51） |
| ML | 18.30  （15.62） | 26.57  （16.98） | 31.90  （19.69） | 75.30  （22.48） | 172.03  （24.10） | 217.67  （24.31） | 315.30  （24.10） | 311.70  （23.94） | 212.87  （23.47） | 128.33  （21.71） | 74.23  （18.88） | 32.67  （15.90） |
| XM | 12.00  （15.07） | 10.15  （16.65） | 16.40  （19.31） | 43.25  （22.47） | 155.55  （24.50） | 228.60  （24.68） | 285.70  （24.26） | 277.90  （24.31） | 181.15  （24.00） | 135.30  （22.16） | 71.40  （18.83） | 20.75  （15.75） |
| MH | 15.33  （14.20） | 16.00  （15.70） | 18.06  （18.44） | 49.78  （21.28） | 162.44  （23.20） | 219.61  （23.21） | 307.78  （22.84） | 305.33  （22.79） | 177.78  （22.34） | 135.28  （20.61） | 64.83  （17.60） | 27.22  （14.71） |
| JH | 19.43  （13.83） | 16.77  （15.34） | 21.30  （18.24） | 52.87  （20.95） | 153.50  （22.87） | 213.27  （22.97） | 306.87  （22.73） | 302.57  （22.58） | 179.20  （22.06） | 140.63  （20.32） | 71.50  （17.26） | 31.43  （14.24） |
