# Supplementary material for: The effects of genetic variation and environmental factors on rhynchophylline and isorhynchophylline in Uncaria macrophylla Wall. from different populations in China
Source: PLoS One. 2018 Jun 28;13(6):e0199259. doi: 10.1371/journal.pone.0199259 (PMC6023176; doi:10.1371/journal.pone.0199259)
Supplement: S2 Table — (DOCX) [file pone.0199259.s002.docx]

**S2 Table. The soil texture information for 9 populations**

| population | cation Exchange capacity | Organic carbon content% | PH | Clay content% | Sand content% |
| --- | --- | --- | --- | --- | --- |
| NP | 10.90 | 1.21 | 5.14 | 41.00 | 35.65 |
| DX | 8.93 | 1.54 | 5.09 | 31.67 | 47.80 |
| FC | 5.91 | 0.96 | 5.15 | 24.91 | 56.00 |
| PB | 16.20 | 2.54 | 4.80 | 48.13 | 26.93 |
| JC | 9.63 | 1.41 | 4.81 | 45.70 | 33.07 |
| ML | 7.50 | 1.22 | 4.75 | 36.50 | 46.50 |
| XM | 9.40 | 1.40 | 5.36 | 44.10 | 30.25 |
| MH | 14.61 | 2.55 | 4.86 | 45.11 | 32.33 |
| JH | 10.00 | 1.42 | 5.05 | 29.00 | 43.43 |
