# Supplementary material for: The effects of genetic variation and environmental factors on rhynchophylline and isorhynchophylline in Uncaria macrophylla Wall. from different populations in China
Source: PLoS One. 2018 Jun 28;13(6):e0199259. doi: 10.1371/journal.pone.0199259 (PMC6023176; doi:10.1371/journal.pone.0199259)
Supplement: S3 Table — (DOCX) [file pone.0199259.s003.docx]

**S3 Table. The information of chemical compounds from 200 individuals**

| individuals | Rhynchophylline(RIN)% | Isorhynchophylline(IRN)% | RIN/IRN | Sum(RIN+ IRN) | RIN/sum% | IRN/sum% |
| --- | --- | --- | --- | --- | --- | --- |
| 1 | 0.1591 | 0.0427 | 3.7249 | 0.2018 | 78.8357 | 21.1643 |
| 2 | 0.2249 | 0.0765 | 2.9412 | 0.3014 | 74.6273 | 25.3727 |
| 3 | 0.1552 | 0.0484 | 3.2095 | 0.2036 | 76.2441 | 23.7559 |
| 4 | 0.1074 | 0.0170 | 6.3032 | 0.1244 | 86.3073 | 13.6927 |
| 5 | 0.1264 | 0.0483 | 2.6147 | 0.1747 | 72.3350 | 27.6650 |
| 6 | 0.1818 | 0.0405 | 4.4852 | 0.2224 | 81.7692 | 18.2308 |
| 7 | 0.2325 | 0.1166 | 1.9935 | 0.3492 | 66.5939 | 33.4061 |
| 8 | 0.1643 | 0.0690 | 2.3823 | 0.2333 | 70.4339 | 29.5661 |
| 9 | 0.1605 | 0.0536 | 2.9944 | 0.2141 | 74.9652 | 25.0348 |
| 10 | 0.1616 | 0.0658 | 2.4546 | 0.2274 | 71.0532 | 28.9468 |
| 11 | 0.1122 | 0.0343 | 3.2709 | 0.1464 | 76.5858 | 23.4142 |
| 12 | 0.2077 | 0.0740 | 2.8057 | 0.2818 | 73.7238 | 26.2762 |
| 13 | 0.2386 | 0.1558 | 1.5318 | 0.3944 | 60.5022 | 39.4978 |
| 14 | 0.1939 | 0.0673 | 2.8836 | 0.2612 | 74.2504 | 25.7496 |
| 15 | 0.1240 | 0.0361 | 3.4312 | 0.1601 | 77.4328 | 22.5672 |
| 16 | 0.0961 | 0.0275 | 3.4935 | 0.1236 | 77.7456 | 22.2544 |
| 17 | 0.1530 | 0.0392 | 3.8981 | 0.1922 | 79.5839 | 20.4161 |
| 18 | 0.2020 | 0.0393 | 5.1387 | 0.2413 | 83.7100 | 16.2900 |
| 19 | 0.2073 | 0.0584 | 3.5486 | 0.2657 | 78.0152 | 21.9848 |
| 20 | 0.1845 | 0.0680 | 2.7133 | 0.2525 | 73.0699 | 26.9301 |
| 21 | 0.2634 | 0.0562 | 4.6877 | 0.3196 | 82.4183 | 17.5817 |
| 22 | 0.1690 | 0.0218 | 7.7428 | 0.1908 | 88.5620 | 11.4380 |
| 23 | 0.1669 | 0.0410 | 4.0703 | 0.2079 | 80.2773 | 19.7227 |
| 24 | 0.2383 | 0.0404 | 5.9042 | 0.2787 | 85.5160 | 14.4840 |
| 25 | 0.1824 | 0.0383 | 4.7658 | 0.2206 | 82.6562 | 17.3438 |
| 26 | 0.1402 | 0.0157 | 8.9320 | 0.1559 | 89.9316 | 10.0684 |
| 27 | 0.1663 | 0.0613 | 2.7114 | 0.2277 | 73.0558 | 26.9442 |
| 28 | 0.2189 | 0.0640 | 3.4222 | 0.2828 | 77.3869 | 22.6131 |
| 29 | 0.2142 | 0.0657 | 3.2577 | 0.2799 | 76.5130 | 23.4870 |
| 30 | 0.1764 | 0.0780 | 2.2612 | 0.2544 | 69.3362 | 30.6638 |
| 31 | 0.1532 | 0.0413 | 3.7073 | 0.1945 | 78.7563 | 21.2437 |
| 32 | 0.2336 | 0.1054 | 2.2163 | 0.3390 | 68.9087 | 31.0913 |
| 33 | 0.2074 | 0.0561 | 3.6939 | 0.2635 | 78.6956 | 21.3044 |
| 34 | 0.2260 | 0.0717 | 3.1532 | 0.2977 | 75.9220 | 24.0780 |
| 35 | 0.1937 | 0.0792 | 2.4467 | 0.2728 | 70.9865 | 29.0135 |
| 36 | 0.1839 | 0.0974 | 1.8878 | 0.2813 | 65.3710 | 34.6290 |
| 37 | 0.1997 | 0.0485 | 4.1155 | 0.2482 | 80.4517 | 19.5483 |
| 38 | 0.2377 | 0.0781 | 3.0429 | 0.3158 | 75.2654 | 24.7346 |
| 39 | 0.2005 | 0.0736 | 2.7242 | 0.2741 | 73.1485 | 26.8515 |
| 40 | 0.1778 | 0.0429 | 4.1451 | 0.2207 | 80.5641 | 19.4359 |
| 41 | 0.2025 | 0.0469 | 4.3228 | 0.2494 | 81.2127 | 18.7873 |
| 42 | 0.1333 | 0.0451 | 2.9558 | 0.1784 | 74.7209 | 25.2791 |
| 43 | 0.2175 | 0.0654 | 3.3241 | 0.2830 | 76.8738 | 23.1262 |
| 44 | 0.1706 | 0.0474 | 3.6009 | 0.2180 | 78.2651 | 21.7349 |
| 45 | 0.2099 | 0.1034 | 2.0304 | 0.3133 | 67.0013 | 32.9987 |
| 46 | 0.2346 | 0.0698 | 3.3625 | 0.3044 | 77.0775 | 22.9225 |
| 47 | 0.3245 | 0.0687 | 4.7257 | 0.3932 | 82.5349 | 17.4651 |
| 48 | 0.2694 | 0.0497 | 5.4258 | 0.3191 | 84.4377 | 15.5623 |
| 49 | 0.2850 | 0.0889 | 3.2079 | 0.3739 | 76.2351 | 23.7649 |
| 50 | 0.2163 | 0.0349 | 6.1953 | 0.2512 | 86.1020 | 13.8980 |
| 51 | 0.3044 | 0.0582 | 5.2292 | 0.3626 | 83.9467 | 16.0533 |
| 52 | 0.2531 | 0.0476 | 5.3227 | 0.3007 | 84.1839 | 15.8161 |
| 53 | 0.1828 | 0.0634 | 2.8812 | 0.2463 | 74.2349 | 25.7651 |
| 54 | 0.3243 | 0.0807 | 4.0189 | 0.4051 | 80.0752 | 19.9248 |
| 55 | 0.2109 | 0.0533 | 3.9574 | 0.2641 | 79.8281 | 20.1719 |
| 56 | 0.1892 | 0.0524 | 3.6130 | 0.2416 | 78.3222 | 21.6778 |
| 57 | 0.1986 | 0.0722 | 2.7519 | 0.2707 | 73.3469 | 26.6531 |
| 58 | 0.1667 | 0.0293 | 5.6822 | 0.1961 | 85.0349 | 14.9651 |
| 59 | 0.1855 | 0.0864 | 2.1474 | 0.2719 | 68.2275 | 31.7725 |
| 60 | 0.1519 | 0.0416 | 3.6501 | 0.1935 | 78.4951 | 21.5049 |
| 61 | 0.2600 | 0.0658 | 3.9525 | 0.3257 | 79.8083 | 20.1917 |
| 62 | 0.2705 | 0.0908 | 2.9789 | 0.3613 | 74.8672 | 25.1328 |
| 63 | 0.3178 | 0.1246 | 2.5505 | 0.4424 | 71.8350 | 28.1650 |
| 64 | 0.1667 | 0.0409 | 4.0796 | 0.2075 | 80.3134 | 19.6866 |
| 65 | 0.2650 | 0.1236 | 2.1438 | 0.3887 | 68.1911 | 31.8089 |
| 66 | 0.1988 | 0.0732 | 2.7166 | 0.2719 | 73.0937 | 26.9063 |
| 67 | 0.1957 | 0.0412 | 4.7554 | 0.2369 | 82.6249 | 17.3751 |
| 68 | 0.2429 | 0.0709 | 3.4256 | 0.3138 | 77.4042 | 22.5958 |
| 69 | 0.2155 | 0.0438 | 4.9183 | 0.2594 | 83.1033 | 16.8967 |
| 70 | 0.2906 | 0.0493 | 5.8922 | 0.3399 | 85.4908 | 14.5092 |
| 71 | 0.1731 | 0.0769 | 2.2513 | 0.2500 | 69.2432 | 30.7568 |
| 72 | 0.2151 | 0.0383 | 5.6227 | 0.2533 | 84.9005 | 15.0995 |
| 73 | 0.2504 | 0.0577 | 4.3381 | 0.3081 | 81.2667 | 18.7333 |
| 74 | 0.2108 | 0.0478 | 4.4107 | 0.2586 | 81.5183 | 18.4817 |
| 75 | 0.2251 | 0.0564 | 3.9895 | 0.2815 | 79.9579 | 20.0421 |
| 76 | 0.2220 | 0.0514 | 4.3209 | 0.2734 | 81.2061 | 18.7939 |
| 77 | 0.0940 | 0.0187 | 5.0190 | 0.1128 | 83.3860 | 16.6140 |
| 78 | 0.2616 | 0.0948 | 2.7597 | 0.3564 | 73.4020 | 26.5980 |
| 79 | 0.1850 | 0.0727 | 2.5448 | 0.2576 | 71.7897 | 28.2103 |
| 80 | 0.1460 | 0.0386 | 3.7803 | 0.1846 | 79.0809 | 20.9191 |
| 81 | 0.2371 | 0.0906 | 2.6157 | 0.3277 | 72.3429 | 27.6571 |
| 82 | 0.2069 | 0.0430 | 4.8153 | 0.2498 | 82.8041 | 17.1959 |
| 83 | 0.1610 | 0.0509 | 3.1645 | 0.2119 | 75.9876 | 24.0124 |
| 84 | 0.1901 | 0.0598 | 3.1786 | 0.2499 | 76.0686 | 23.9314 |
| 85 | 0.1420 | 0.0298 | 4.7714 | 0.1718 | 82.6730 | 17.3270 |
| 86 | 0.1639 | 0.0285 | 5.7575 | 0.1924 | 85.2015 | 14.7985 |
| 87 | 0.2010 | 0.0281 | 7.1494 | 0.2291 | 87.7292 | 12.2708 |
| 88 | 0.1494 | 0.0552 | 2.7042 | 0.2047 | 73.0040 | 26.9960 |
| 89 | 0.2081 | 0.0516 | 4.0351 | 0.2596 | 80.1395 | 19.8605 |
| 90 | 0.1913 | 0.0490 | 3.9037 | 0.2403 | 79.6071 | 20.3929 |
| 91 | 0.1734 | 0.0383 | 4.5337 | 0.2117 | 81.9289 | 18.0711 |
| 92 | 0.1820 | 0.0932 | 1.9527 | 0.2751 | 66.1325 | 33.8675 |
| 93 | 0.1635 | 0.0621 | 2.6320 | 0.2256 | 72.4673 | 27.5327 |
| 94 | 0.1508 | 0.0304 | 4.9586 | 0.1813 | 83.2177 | 16.7823 |
| 95 | 0.1556 | 0.0339 | 4.5836 | 0.1896 | 82.0905 | 17.9095 |
| 96 | 0.1317 | 0.0318 | 4.1422 | 0.1636 | 80.5532 | 19.4468 |
| 97 | 0.1772 | 0.0350 | 5.0673 | 0.2122 | 83.5182 | 16.4818 |
| 98 | 0.2103 | 0.0426 | 4.9330 | 0.2530 | 83.1451 | 16.8549 |
| 99 | 0.1615 | 0.0413 | 3.9101 | 0.2028 | 79.6339 | 20.3661 |
| 100 | 0.1511 | 0.0424 | 3.5629 | 0.1935 | 78.0841 | 21.9159 |
| 101 | 0.1548 | 0.0339 | 4.5654 | 0.1887 | 82.0318 | 17.9682 |
| 102 | 0.1593 | 0.0792 | 2.0110 | 0.2385 | 66.7890 | 33.2110 |
| 103 | 0.2255 | 0.0549 | 4.1069 | 0.2804 | 80.4187 | 19.5813 |
| 104 | 0.1915 | 0.0484 | 3.9563 | 0.2400 | 79.8236 | 20.1764 |
| 105 | 0.2664 | 0.0360 | 7.4065 | 0.3023 | 88.1045 | 11.8955 |
| 106 | 0.1686 | 0.0371 | 4.5406 | 0.2057 | 81.9514 | 18.0486 |
| 107 | 0.2049 | 0.0477 | 4.2932 | 0.2526 | 81.1077 | 18.8923 |
| 108 | 0.1524 | 0.0451 | 3.3821 | 0.1975 | 77.1799 | 22.8201 |
| 109 | 0.1696 | 0.0487 | 3.4863 | 0.2183 | 77.7098 | 22.2902 |
| 110 | 0.3158 | 0.0697 | 4.5320 | 0.3854 | 81.9233 | 18.0767 |
| 111 | 0.1630 | 0.0755 | 2.1581 | 0.2385 | 68.3353 | 31.6647 |
| 112 | 0.2039 | 0.1179 | 1.7294 | 0.3219 | 63.3622 | 36.6378 |
| 113 | 0.1852 | 0.0514 | 3.6015 | 0.2367 | 78.2677 | 21.7323 |
| 114 | 0.1973 | 0.0585 | 3.3691 | 0.2558 | 77.1122 | 22.8878 |
| 115 | 0.2175 | 0.1008 | 2.1574 | 0.3183 | 68.3284 | 31.6716 |
| 116 | 0.2319 | 0.0488 | 4.7509 | 0.2807 | 82.6113 | 17.3887 |
| 117 | 0.2965 | 0.0953 | 3.1122 | 0.3917 | 75.6821 | 24.3179 |
| 118 | 0.2009 | 0.0556 | 3.6139 | 0.2565 | 78.3264 | 21.6736 |
| 119 | 0.2050 | 0.0739 | 2.7756 | 0.2789 | 73.5144 | 26.4856 |
| 120 | 0.1808 | 0.0745 | 2.4287 | 0.2553 | 70.8341 | 29.1659 |
| 121 | 0.1906 | 0.0471 | 4.0482 | 0.2377 | 80.1908 | 19.8092 |
| 122 | 0.2940 | 0.2130 | 1.3799 | 0.5070 | 57.9819 | 42.0181 |
| 123 | 0.2024 | 0.0340 | 5.9544 | 0.2364 | 85.6206 | 14.3794 |
| 124 | 0.1854 | 0.0950 | 1.9521 | 0.2804 | 66.1261 | 33.8739 |
| 125 | 0.1675 | 0.0468 | 3.5772 | 0.2144 | 78.1528 | 21.8472 |
| 126 | 0.1763 | 0.0418 | 4.2149 | 0.2182 | 80.8240 | 19.1760 |
| 127 | 0.1764 | 0.0735 | 2.3986 | 0.2499 | 70.5758 | 29.4242 |
| 128 | 0.1972 | 0.0364 | 5.4216 | 0.2336 | 84.4275 | 15.5725 |
| 129 | 0.1725 | 0.0896 | 1.9254 | 0.2621 | 65.8162 | 34.1838 |
| 130 | 0.1483 | 0.0218 | 6.8052 | 0.1701 | 87.1880 | 12.8120 |
| 131 | 0.3458 | 0.0815 | 4.2422 | 0.4273 | 80.9239 | 19.0761 |
| 132 | 0.1739 | 0.0507 | 3.4321 | 0.2245 | 77.4373 | 22.5627 |
| 133 | 0.2750 | 0.0977 | 2.8144 | 0.3728 | 73.7834 | 26.2166 |
| 134 | 0.1959 | 0.0587 | 3.3380 | 0.2546 | 76.9477 | 23.0523 |
| 135 | 0.2019 | 0.0874 | 2.3090 | 0.2893 | 69.7793 | 30.2207 |
| 136 | 0.1617 | 0.0891 | 1.8137 | 0.2508 | 64.4594 | 35.5406 |
| 137 | 0.1598 | 0.0514 | 3.1089 | 0.2112 | 75.6625 | 24.3375 |
| 138 | 0.1694 | 0.0532 | 3.1824 | 0.2226 | 76.0903 | 23.9097 |
| 139 | 0.1858 | 0.0326 | 5.6984 | 0.2185 | 85.0711 | 14.9289 |
| 140 | 0.1410 | 0.0476 | 2.9594 | 0.1886 | 74.7439 | 25.2561 |
| 141 | 0.1905 | 0.0370 | 5.1524 | 0.2274 | 83.7463 | 16.2537 |
| 142 | 0.1401 | 0.0297 | 4.7134 | 0.1698 | 82.4974 | 17.5026 |
| 143 | 0.2205 | 0.0823 | 2.6792 | 0.3028 | 72.8203 | 27.1797 |
| 144 | 0.2576 | 0.0705 | 3.6522 | 0.3281 | 78.5050 | 21.4950 |
| 145 | 0.2286 | 0.0667 | 3.4267 | 0.2954 | 77.4099 | 22.5901 |
| 146 | 0.1649 | 0.0413 | 3.9946 | 0.2062 | 79.9783 | 20.0217 |
| 147 | 0.2244 | 0.0699 | 3.2105 | 0.2943 | 76.2500 | 23.7500 |
| 148 | 0.1877 | 0.0605 | 3.1039 | 0.2482 | 75.6331 | 24.3669 |
| 149 | 0.1705 | 0.0451 | 3.7810 | 0.2156 | 79.0840 | 20.9160 |
| 150 | 0.2426 | 0.0892 | 2.7185 | 0.3318 | 73.1075 | 26.8925 |
| 151 | 0.1515 | 0.0442 | 3.4251 | 0.1957 | 77.4015 | 22.5985 |
| 152 | 0.1432 | 0.0451 | 3.1728 | 0.1884 | 76.0353 | 23.9647 |
| 153 | 0.1926 | 0.0455 | 4.2311 | 0.2382 | 80.8836 | 19.1164 |
| 154 | 0.2548 | 0.0879 | 2.8987 | 0.3427 | 74.3504 | 25.6496 |
| 155 | 0.1977 | 0.0316 | 6.2532 | 0.2294 | 86.2130 | 13.7870 |
| 156 | 0.2152 | 0.0628 | 3.4255 | 0.2780 | 77.4037 | 22.5963 |
| 157 | 0.1828 | 0.0484 | 3.7755 | 0.2313 | 79.0599 | 20.9401 |
| 158 | 0.2368 | 0.0803 | 2.9504 | 0.3171 | 74.6861 | 25.3139 |
| 159 | 0.1721 | 0.0506 | 3.3999 | 0.2227 | 77.2724 | 22.7276 |
| 160 | 0.1814 | 0.0678 | 2.6733 | 0.2492 | 72.7767 | 27.2233 |
| 161 | 0.1793 | 0.0514 | 3.4905 | 0.2306 | 77.7310 | 22.2690 |
| 162 | 0.2582 | 0.0883 | 2.9231 | 0.3466 | 74.5097 | 25.4903 |
| 163 | 0.2159 | 0.0387 | 5.5770 | 0.2546 | 84.7955 | 15.2045 |
| 164 | 0.1543 | 0.0530 | 2.9109 | 0.2073 | 74.4301 | 25.5699 |
| 165 | 0.2146 | 0.0735 | 2.9202 | 0.2881 | 74.4908 | 25.5092 |
| 166 | 0.2581 | 0.1294 | 1.9939 | 0.3875 | 66.5986 | 33.4014 |
| 167 | 0.2705 | 0.0906 | 2.9866 | 0.3611 | 74.9162 | 25.0838 |
| 168 | 0.3241 | 0.1217 | 2.6641 | 0.4458 | 72.7081 | 27.2919 |
| 169 | 0.2668 | 0.1071 | 2.4899 | 0.3739 | 71.3459 | 28.6541 |
| 170 | 0.1943 | 0.0537 | 3.6215 | 0.2480 | 78.3620 | 21.6380 |
| 171 | 0.1822 | 0.0433 | 4.2056 | 0.2255 | 80.7899 | 19.2101 |
| 172 | 0.2025 | 0.0583 | 3.4719 | 0.2609 | 77.6381 | 22.3619 |
| 173 | 0.1529 | 0.0235 | 6.5050 | 0.1764 | 86.6755 | 13.3245 |
| 174 | 0.1365 | 0.0533 | 2.5631 | 0.1898 | 71.9347 | 28.0653 |
| 175 | 0.2178 | 0.0549 | 3.9699 | 0.2727 | 79.8788 | 20.1212 |
| 176 | 0.1913 | 0.0448 | 4.2720 | 0.2361 | 81.0319 | 18.9681 |
| 177 | 0.1789 | 0.0630 | 2.8423 | 0.2419 | 73.9739 | 26.0261 |
| 178 | 0.1582 | 0.0348 | 4.5432 | 0.1930 | 81.9600 | 18.0400 |
| 179 | 0.2650 | 0.0680 | 3.8976 | 0.3330 | 79.5819 | 20.4181 |
| 180 | 0.2223 | 0.0471 | 4.7240 | 0.2694 | 82.5298 | 17.4702 |
| 181 | 0.2468 | 0.0715 | 3.4507 | 0.3183 | 77.5316 | 22.4684 |
| 182 | 0.1796 | 0.0442 | 4.0595 | 0.2238 | 80.2351 | 19.7649 |
| 183 | 0.1553 | 0.0380 | 4.0807 | 0.1933 | 80.3175 | 19.6825 |
| 184 | 0.2463 | 0.0811 | 3.0364 | 0.3275 | 75.2256 | 24.7744 |
| 185 | 0.2199 | 0.0640 | 3.4335 | 0.2839 | 77.4445 | 22.5555 |
| 186 | 0.1844 | 0.0536 | 3.4405 | 0.2380 | 77.4798 | 22.5202 |
| 187 | 0.2073 | 0.0738 | 2.8084 | 0.2811 | 73.7426 | 26.2574 |
| 188 | 0.1735 | 0.0630 | 2.7529 | 0.2365 | 73.3542 | 26.6458 |
| 189 | 0.2554 | 0.0725 | 3.5209 | 0.3279 | 77.8806 | 22.1194 |
| 190 | 0.2538 | 0.0766 | 3.3124 | 0.3304 | 76.8113 | 23.1887 |
| 191 | 0.2104 | 0.0843 | 2.4962 | 0.2948 | 71.3974 | 28.6026 |
| 192 | 0.2000 | 0.0612 | 3.2694 | 0.2612 | 76.5777 | 23.4223 |
| 193 | 0.2413 | 0.0790 | 3.0534 | 0.3203 | 75.3296 | 24.6704 |
| 194 | 0.2383 | 0.0785 | 3.0367 | 0.3168 | 75.2273 | 24.7727 |
| 195 | 0.2022 | 0.0565 | 3.5822 | 0.2587 | 78.1763 | 21.8237 |
| 196 | 0.1729 | 0.0452 | 3.8240 | 0.2181 | 79.2703 | 20.7297 |
| 197 | 0.2788 | 0.1110 | 2.5109 | 0.3898 | 71.5177 | 28.4823 |
| 198 | 0.1600 | 0.0575 | 2.7803 | 0.2175 | 73.5474 | 26.4526 |
| 199 | 0.1505 | 0.0289 | 5.2044 | 0.1794 | 83.8823 | 16.1177 |
| 200 | 0.1755 | 0.0397 | 4.4241 | 0.2152 | 81.5637 | 18.4363 |
