# Supplementary material for: The effects of genetic variation and environmental factors on rhynchophylline and isorhynchophylline in Uncaria macrophylla Wall. from different populations in China
Source: PLoS One. 2018 Jun 28;13(6):e0199259. doi: 10.1371/journal.pone.0199259 (PMC6023176; doi:10.1371/journal.pone.0199259)
Supplement: S4 Table — (DOCX) [file pone.0199259.s004.docx]

**S4 Table. ISSR primer screening**

| Primer code | Sequence(5′—3′) | Annealing temperature(℃) | No. of amplified bands | No. of polymorphic bands | The percentage of polymorphic loci |
| --- | --- | --- | --- | --- | --- |
| UBC807 | AGAGAGAGAGAGAGAGT | 55 | 17 | 17 | 100% |
| UBC808 | AGAGAGAGAGAGAGAGC | 57 | 18 | 18 | 100% |
| UBC809 | AGAGAGAGAGAGAGAGG | 57 | 22 | 22 | 100% |
| UBC812 | GAGAGAGAGAGAGAGAA | 55 | 17 | 17 | 100% |
| UBC835 | AGAGAGAGAGAGAGAGYC | 57 | 26 | 25 | 96.15% |
| UBC840 | GAGAGAGAGAGAGAGAYT | 55 | 20 | 20 | 100% |
| UBC844 | CTCTCTCTCTCTCTCTRC | 57 | 16 | 16 | 100% |
| UBC846 | CACACACACACACACART | 55 | 24 | 22 | 91.67% |
| UBC847 | CACACACACACACACARC | 57 | 17 | 16 | 94.11% |
| UBC848 | CACACACACACACACARG | 57 | 24 | 22 | 91.67% |
| Total |  |  | 201 | 195 | 97.01% |
