# Supplementary material for: The effects of genetic variation and environmental factors on rhynchophylline and isorhynchophylline in Uncaria macrophylla Wall. from different populations in China
Source: PLoS One. 2018 Jun 28;13(6):e0199259. doi: 10.1371/journal.pone.0199259 (PMC6023176; doi:10.1371/journal.pone.0199259)
Supplement: S5 Table — (DOCX) [file pone.0199259.s005.docx]

**S5 Table. Genetic diversity index of the 9 populations**

| Population | Na(Observed number of alleles) | Ne(Effective number of alleles) | H(Nei's (1973) gene diversity) | I (Shannon's Information index) | Polymorphic  sites | Proportion of polymorphism |
| --- | --- | --- | --- | --- | --- | --- |
| NP | 1.6816±0.4670 | 1.3368±0.3521 | 0.2026±0.1910 | 0.3094±0.2709 | 137 | 68.16% |
| DX | 1.6219±0.4861 | 1.2858±0.3377 | **0.1752±0.1827** | **0.2723±0.2611** | 125 | **62.19%** |
| PX | 1.6517±0.4776 | 1.3040±0.3408 | 0.1860±0.1838 | 0.2884±0.2616 | 131 | 65.17% |
| PB | 1.6915±0.4630 | 1.3140±0.3492 | 0.1910±0.1842 | 0.2970±0.2593 | 139 | 69.15% |
| JC | 1.7512±0.4334 | 1.3296±0.3376 | **0.2031±0.1799** | **0.3167±0.2529** | 151 | **75.12%** |
| ML | 1.7512±0.4334 | 1.3437±0.3424 | **0.2106±0.1812** | **0.3266±0.2545** | 151 | **75.12%** |
| XM | 1.6617±0.4743 | 1.3033±0.3394 | 0.1857±0.1840 | 0.2879±0.2620 | 133 | 66.17% |
| MH | 1.6517±0.4776 | 1.3352±0.3714 | 0.1969±0.1976 | 0.2990±0.2777 | 131 | 65.17% |
| JH | 1.6468±0.4792 | 1.3225±0.3672 | 0.1905±0.1953 | 0.2903±0.2756 | 130 | 64.68% |
| Total | 1.9701±0.1706 | 1.3870±0.3197 | 0.2424±0.1623 | 0.3820±0.2161 | 195 | 97.01% |
