# Supplementary material for: The effects of genetic variation and environmental factors on rhynchophylline and isorhynchophylline in Uncaria macrophylla Wall. from different populations in China
Source: PLoS One. 2018 Jun 28;13(6):e0199259. doi: 10.1371/journal.pone.0199259 (PMC6023176; doi:10.1371/journal.pone.0199259)
Supplement: S6 Table — (DOCX) [file pone.0199259.s006.docx]

**S6 Table**. **The correlation between chemical compounds and other environment indexes in 9 populations**.

| Variables | Latitude | Longitude | Altitude(m) | Variable coefficient of seasonal precipitation | Variation range of mean annual temperature（℃） | Mean annual precipitation（mm） | Mean annual temperature（℃） |
| --- | --- | --- | --- | --- | --- | --- | --- |
| RIN% | -0.387(0.303) | 0.013(0.974) | -0.077(0.844) | **-0.693(0.038)** | -0.172(0.658) | -0.540(0.134) | 0.210(0.588) |
| IRN% | -0.359(0.343) | -0.140(0.719) | 0.0910(0.815) | -0.299(0.434) | -0.096(0.807) | -0.587(0.097) | 0.042(0.914) |
| RIN/IRN | -0.053(0.892) | 0.168(0.665) | -0.183(0.638) | -0.491(0.180) | -0.110(0.778) | 0.125(0.748) | 0.184(0.635) |

Values in bold are different from 0 with a significant level, alpha=0.05. P-values are listed in parentheses.
