# Supplementary material for: The effects of genetic variation and environmental factors on rhynchophylline and isorhynchophylline in Uncaria macrophylla Wall. from different populations in China
Source: PLoS One. 2018 Jun 28;13(6):e0199259. doi: 10.1371/journal.pone.0199259 (PMC6023176; doi:10.1371/journal.pone.0199259)
Supplement: S7 Table — (DOCX) [file pone.0199259.s007.docx]

**S7 Table. The correlation between chemical compounds and Monthly temperature in 9 populations**

| Variables | 1 | 2 | 3 | 4 | 5 | 6 | 7 | 8 | 9 | 10 | 11 | 12 |
| --- | --- | --- | --- | --- | --- | --- | --- | --- | --- | --- | --- | --- |
| RIN% | 0.327  (0.390) | 0.249  (0.518) | 0.378  (0.316) | 0.235  (0.542) | 0.183  (0.637) | 0.109  (0.780) | 0.065  (0.869) | 0.059  (0.880) | 0.111  (0.777) | 0.158  (0.685) | 0.275  (0.473) | 0.358  (0.344) |
| IRN% | 0.277  (0.471) | 0.287  (0.454) | 0.363  (0.337) | 0.158  (0.685) | 0.017  (0.965) | -0.058  (0.881) | -0.086  (0.825) | -0.108  (0.781) | -0.073  (0.851) | -0.026  (0.947) | 0.093  (0.811) | 0.230  (0.552) |
| RIN/IRN% | 0.083  (0.832) | -0.034  (0.932) | 0.020  (0.960) | 0.070  (0.859) | 0.174  (0.653) | 0.178  (0.647) | 0.158  (0.686) | 0.180  (0.643) | 0.201  (0.604) | 0.208  (0.591) | 0.215  (0.578) | 0.151  (0.698) |
| Sum | 0.332  (0.383) | 0.271  (0.481) | 0.394  (0.293) | 0.230  (0.551) | 0.154  (0.692) | 0.076  (0.846) | 0.033  (0.933) | 0.023  (0.953) | 0.073  (0.852) | 0.123  (0.753) | 0.247  (0.522) | 0.346  (0.361) |
| RIN/Sum% | 0.113  (0.772) | -0.004  (0.991) | 0.044  (0.910) | 0.065  (0.869) | 0.157  (0.687) | 0.153  (0.694) | 0.129  (0.741) | 0.152  (0.697) | 0.178  (0.647) | 0.195  (0.614) | 0.214  (0.581) | 0.161  (0.678) |
| IRN/Sum% | -0.113  (0.772) | 0.004  (0.991) | -0.044  (0.910) | -0.065  (0.869) | -0.157  (0.687) | -0.153  (0.694) | -0.129  (0.741) | -0.152  (0.697) | -0.178  (0.647) | -0.195  (0.614) | -0.214  (0.581) | -0.161  (0.678) |

Values in bold are different from 0 with a significant level, alpha=0.05. P-values are listed in parentheses.
