# Supplementary material for: The effects of genetic variation and environmental factors on rhynchophylline and isorhynchophylline in Uncaria macrophylla Wall. from different populations in China
Source: PLoS One. 2018 Jun 28;13(6):e0199259. doi: 10.1371/journal.pone.0199259 (PMC6023176; doi:10.1371/journal.pone.0199259)
Supplement: S8 Table — (DOCX) [file pone.0199259.s008.docx]

**S8 Table. The correlation between chemical compounds and soil texture in 9 populations**

| Variables | cation Exchange capacity | Organic carbon content% | PH | Clay content% | Sand content% |
| --- | --- | --- | --- | --- | --- |
| RIN% | 0.044(0.911) | 0.255(0.507) | -0.219(0.571) | -0.391(0.298) | 0.356(0.347) |
| IRN% | 0.215(0.578) | 0.258(0.503) | -0.108(0.782) | -0.178(0.647) | 0.112(0.775) |
| RIN/IRN% | -0.224(0.562) | -0.002(0.995) | -0.184(0.636) | -0.251(0.516) | 0.300(0.433) |
| Sum(RIN+IRN) | 0.089(0.821) | 0.271(0.481) | -0.205(0.597) | -0.360(0.341) | 0.316(0.408) |
| RIN/Sum% | -0.206(0.594) | 0.027(0.944) | -0.191(0.622) | -0.249(0.518) | 0.293(0.444) |
| IRN/Sum% | 0.206(0.594) | -0.027(0.944) | 0.191(0.622) | 0.249(0.518) | -0.293(0.444) |

Values in bold are different from 0 with a significant level, alpha=0.05. P-values are listed in parentheses.
